# Recent advances in understanding the spectrum of genetic determinants of lipoprotein(a) levels

**DOI:** 10.1097/MOL.0000000000001030

**Published:** 2026-02-06

**Authors:** Stefan Coassin

**Affiliations:** Institute of Genetic Epidemiology, Medical University of Innsbruck, Innsbruck, Austria

**Keywords:** cardiovascular genetics, genomic technologies, KIV-2 copy number variation, lipoprotein(a), long-read sequencing

## Abstract

**Purpose of review:**

Our understanding of the genetic regulation of lipoprotein(a) [Lp(a)] is hindered by the complex structure of the *LPA* gene, limited non-European datasets and its elusive cellular receptor(s). This review summarizes recent efforts and advances providing new insights on its genetic architecture, variability across ancestries and regulators beyond the *LPA* gene.

**Recent findings:**

Impressive advances in DNA sequencing and bioinformatics now resolve *LPA* variants and kringle IV-type 2 copy number at scale. This provides new reference datasets and enables tools that unlock hidden variation also from already available sequencing datasets. In parallel, genetic studies broaden our understanding of the regulation of Lp(a) across ancestries and improve genetic risk scores. Finally, while recent studies implicate new mechanisms for Lp(a) uptake, upcoming genome-wide gene knockout screens allow comprehensive, agnostic scans for regulators and receptors. Puzzlingly, this still converges on the LDL receptor, whose exact role in Lp(a) uptake remains enigmatic.

**Summary:**

Technological advances establish a foundation for more accurate genetic risk assessment across ancestries. These advances are enhancing our understanding of Lp(a) regulation and build a framework for future integrative genetic studies, which may shed new light on the evolution of the Lp(a) trait, adding important context for its physiological and clinical relevance.

## INTRODUCTION

Lipoprotein(a) [Lp(a)] is as a frequent, independent and causal risk factor for multiple cardiovascular diseases (CVDs), with a clear dose-dependent relationship [1,2^▪▪^,3^▪^,^4^,^5^^▪▪^] and a wide range of potential pathophysiological effects [6^▪^]. On a per-particle basis, Lp(a) is approximately six times more atherogenic than LDL [7], and very high concentrations are associated with a three-fold increase in CVD risk [8^▪^,^9^]. 

**Box 1:**
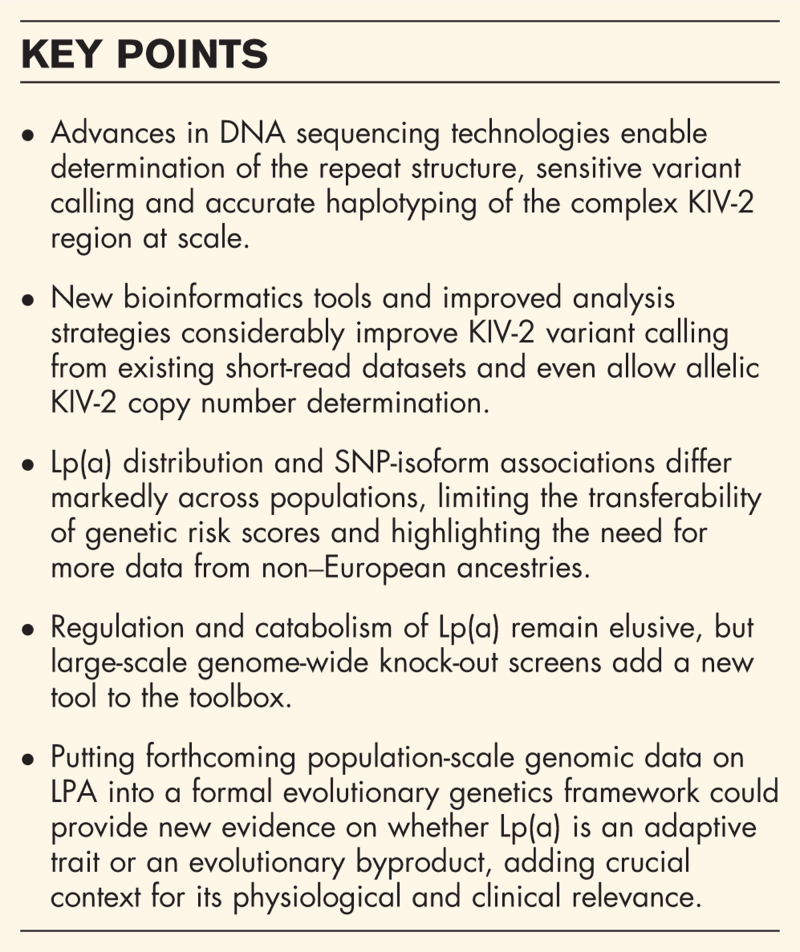
no caption available

**FIGURE 1:**
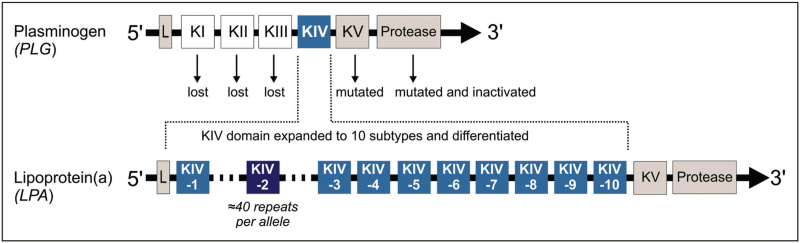
Domain structure and evolution of the lipoprotein(a) gene *LPA* from plasminogen. *LPA* evolved from duplication and remodeling of plasminogen. Each kringle domain consists of two short exons of mostly 160 and 184 bp, spaced by a mostly ≈4 kb long intron, and is linked to the next kringle domain by an ≈1.2 kb intron. All KIV coding sequences are highly similar, with homology extending also into the introns [18]. L: Leader sequence.

However, even after 60 years of research, Lp(a) remains an enigmatic trait with many unanswered questions [10^▪^,11^▪^,^12^]. Individual Lp(a) concentrations range from less than 0.1 to more than 300 mg/dl (approximately <0.2 to 750 nmol/l), with median levels and distribution showing marked differences between and within ancestries [1,13,14]. Yet, more than 90% of Lp(a) variance is controlled by the *LPA* gene locus [15,16], making Lp(a) a highly oligogenic, if not effectively monogenic, trait. *LPA* encodes apolipoprotein(a) [apo(a)], the characteristic structural protein of the Lp(a) particle, and evolved through duplication and remodeling of the plasminogen gene (*PLG*) [17^▪▪^]. It consists of 10 highly similar kringle IV domains (apo(a) domains KIV-1 to KIV-10), one kringle V (KV) and an inactive protease domain [17^▪▪^] (Fig. 1). The KIV-2 domain exists in at least three subtypes (KIV-2A, KIV-2B and KIV-2C) distinguished by three exonic single nucleotide polymorphisms (SNPs) and is highly copy number-variable, presenting 1 to ≈40 copies per allele, spanning up to 70% of the *LPA *coding region and resulting in ≈40 protein isoforms [19].

The KIV-2 copy number explains ≈30-60% of Lp(a) variance via an inverse correlation with endoplasmatic reticulum (ER) transit time [17^▪▪^]. Typically, most circulating Lp(a) originates from the apo(a) isoform with less KIV-2 copies [20], but the relationship between isoform size and Lp(a) plasma concentration is complex [19]. Low-molecular weight (LMW) isoforms with 22 KIV units or less express five to 10 times higher *median* Lp(a) levels than larger ones [19,20], but isoforms of identical size may still express widely differing *individual* Lp(a) levels, ranging from ≈0 to more than 200 mg/dl [21]. This pronounced variance is genetically determined [15,21,22], but the causal variants remained elusive for a long time because of the structural complexity and intricate linkage disequilibrium patterns of the *LPA* gene [19]. Only recently advances in sequencing and bioinformatics uncovered numerous functional SNPs that explain much of the Lp(a) variance in same-sized isoforms but were previously hidden in the KIV-2 region[18,23–27].

Yet, deciphering the *LPA* gene and the Lp(a) trait remains a challenge. This review highlights recent technological advances in mapping variation in the *LPA* gene, understanding its genetic architecture beyond European populations, and uncovering the genetic determinants of Lp(a) concentrations.

## NEW TECHNOLOGIES: LIPIDOLOGY MEETS GENOMICS

**FIGURE 2:**
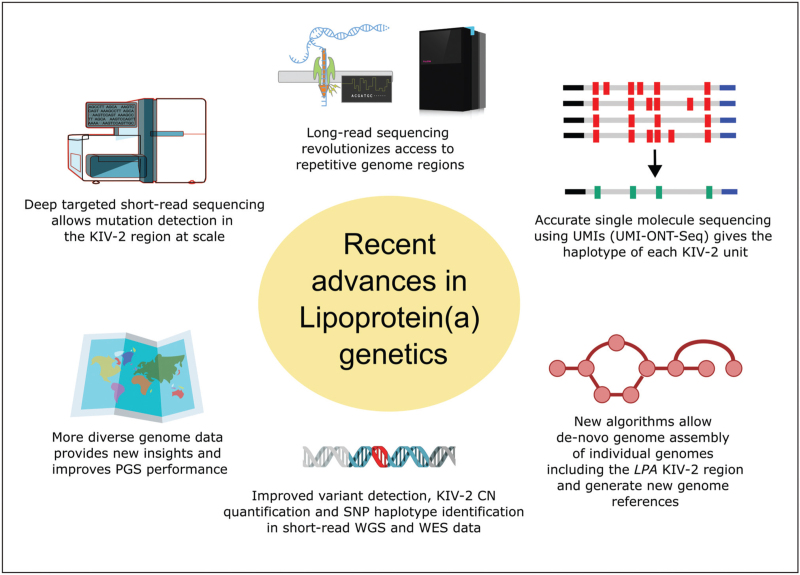
Recent advances in genomics that benefit genetic research on lipoprotein(a). The targeted amplification of all KIV-2 units using universal primers, followed by deep short-read sequencing and mutation detection in read subfractions (termed “KIV-2 batch sequencing” [18]) has long been the cornerstone of *LPA* mutation analysis (represented top left). This review describes multiple new technological advances that benefit genetic Lipoprotein(a) research. The figure uses icons from NIAID NIH BioArt, Bioicons.com and svgrepo.com. See the Acknowledgements section for attributions.

Conventional short-read sequencing (NGS) is poorly suited for resolving genome regions like *LPA* (Supplementary Table 1) and the complex variation patterns in the KIV-2 region also escape current genomic data standards, excluding *LPA* from benchmark datasets and hindering method development [28,29]. These challenges have sparked considerable interest in *LPA* within the genomics community, and – largely unnoticed by the lipidology field – *LPA* has evolved from a cardiovascular curiosity into a locus at the forefront of genomics research [30^▪^,^31^–^35^] (Fig. 2).

Long-read sequencing (LRS) technologies generate reads spanning tens or even hundreds of kilobases and have transformed genomics [36^▪^], enabling the creation of the first complete human genome reference sequence (T2T-CHM13) [37]. This new reference sequence contains an *LPA* gene with 23 KIV-2 units, compared with six in the previous reference hg38, and indicates that the KIV-2B units, which complicate KIV-2 variant calling in ≈80% of the individuals [38^▪^], cluster at the end of the KIV-2 array rather than being interspersed as in hg38 (confirming an earlier preprint [39]). Additional insights into *LPA* structure and variability are promised also by hundreds of forthcoming high-quality long-read genomes [40,41^▪^,^42^,43^▪^]. For example, Gustafson *et al.* [41^▪^] reported that 93% of their LRS genome assemblies achieved full contiguity in *LPA,* though without providing orthogonal validation or further details. Similarly, a recent preprint describes a nearly error-free diploid benchmark genome with a likely gap-free, fully resolved assembly of both *LPA* alleles [44^▪^]. The two alleles differ by 55 kb, that is 10 KIV-2 units, and are accessible via a dedicated UCSC Genome browser data hub. These new reference datasets enable new analysis tools for available data, such as the variant calling tool *Locityper,* which determines KIV-2 SNP haplotypes from any sequencing technology by selecting the haplotype pair in the reference data that best explains the observed sequencing reads [45^▪^]. As LRS reference datasets grow in size and population diversity, such tools are poised to augment available short-read datasets. Of note, even LRS reads rarely span the full KIV-2 region at high coverage, particularly in large alleles, so full reconstruction will still require computationally intensive de-novo assembly.

For more targeted analyses, we have recently introduced a scalable method for direct KIV-2 sequencing and haplotyping, which combines long-read nanopore sequencing with molecular barcode-based, single molecule-level error correction (UMI-ONT-Seq) [46^▪^]. This produced highly accurate consensus sequences for each KIV-2 unit, which confirmed, for example, that the two major European Lp(a)-lowering variants KIV-2 4925G>A [23] and KIV-2 4733G>A [24] occur on different haplotypes [46^▪^]. By counting unique haplotypes and adjusting for coverage, UMI-ONT-Seq also estimates total KIV-2 copy number (CN) with accuracy comparable to digital PCR [46^▪^], which already outperforms KIV-2 CN quantification by qPCR [47]. While assessing only the total KIV-2 copy number has important limitations [48,49], large biobanks have used sequencing-based KIV-2 copy number quantification as an alternative for apo(a) sizing by Western blot, offsetting its imprecision with statistical power [25,50,51]. Generally, sequencing-based KIV-2 copy number quantification explains similar Lp(a) variance as KIV-2 copy number assessment by qPCR (*R*^2^ ≈ 20–30%) but showing better parent-offspring concordance [50].

## EXISTING DATA, NEW TRICKS: UNLOCKING *LPA* FROM SHORT-READ SEQUENCING DATA

Since most large biobanks provide only short-read data, various groups have sought to improve the utility of this data for *LPA* genetics by developing new analysis methods and reanalyzing available NGS data [38^▪^,52^▪^,^53^^▪▪^].

**FIGURE 3:**
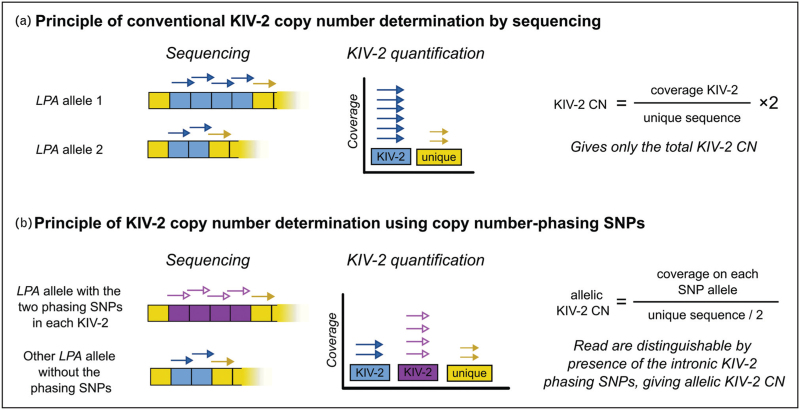
Determination of the KIV-2 copy number (CN) and determination of the allele sizes by sequencing. A. In conventional KIV-2 CN determination, the sequencing coverage on the KIV-2 is normalized to that of one or more unique genome regions. B. When present, the KIV-2 CN phasing SNPs [53^▪▪^] occur in every KIV-2 unit of the corresponding gene allele. In heterozygous individuals (≈40–50% of the population), this allows assigning reads to the two alleles. Normalizing read counts for each SNP allele against a unique sequence provides the KIV-2 CN of each allele. The regions used for normalization can be the nonrepetitive part of *LPA*, as in KILDA [54^▪^], or multiple genome regions, as in DRAGEN [53^▪▪^]. Note that the figure shows only a naïve calculation model for illustration purposes, while real-world data requires more advanced statistics and normalization.

Behera *et al.* [53^▪▪^] recently identified two intronic KIV-2 SNPs that, when present, occur in every repeat of the gene allele. In heterozygous individuals (40–52%, depending on ancestry) and after proper normalization against diploid genome regions, these SNPs allow determining the KIV-2 copy number of each allele with notable accuracy, as the sequencing coverage on the two SNP alleles reflects the number of KIV-2 units on each allele (Fig. 3) [53^▪▪^]. The two SNPs occur in all five major ancestry groups of the 1000 Genomes project, suggesting that they may trace back to very ancestral *LPA* alleles and that recombination within KIV-2 is rare, which is consistent with the long haplotypes observed by Lanktree *et al.* [55]. Behera *et al.* [53^▪▪^] successfully reproduced known ancestry-specific allele size distributions, replicated known SNP-isoform associations (e.g. for rs41272110 [27], rs3798220 [56], and rs10455872 [56]) and the allele size estimates in 60 trios correlated strongly (*R*^2^ > 0.997) with Bionano Optical Mapping data (an optical copy number counting method), supporting validity of the method. This approach has been recently applied also to 8,351 short-read genomes from the GENESIS-HD study (Betschart *et al.* [52^▪^]), again replicating known SNP-isoform associations and complex linkage patterns. Unfortunately, the algorithm has been integrated into Illumina's commercial platforms (BaseSpace, DRAGEN) and submitted as intellectual property [57], limiting its accessibility. Of note, Betschart *et al.* [52^▪^] and Molitor *et al.* [54^▪^] found that other sequencing coverage-based KIV-2 copy number callers perform equally well at least for total KIV 2 copy number determination.

Accurate variant detection in the KIV-2 from whole-genome or whole-exome data, as provided by large biobanks, is complicated by the high homology between the kringle domains, which causes misaligned reads and spurious variant calls [38^▪^]. Our group has observed recently that the best read-mapping strategy strongly depends on presence of KIV-2B units in the analyzed individual genome (see also explanations in Supplementary Table 1) and identified a SNP outside the KIV-2 that predicts KIV-2B presence [38^▪^]. Building on this observation, we developed a scalable open-source tool that selectively remaps short-read KIV-2 reads data, choosing the best alignment strategy dynamically based on the individual genotype. This doubled variant calling accuracy in the KIV-2 region and detected more than 700 high confidence KIV-2 mutations in ≈199 000 UK Biobank samples [38^▪^]. Unfortunately, this KIV-2B tag SNP approach is currently applicable only to Europeans, highlighting the need for more data from other ancestries.

## GENETIC EFFECTS ACROSS ANCESTRIES AND THEIR IMPACT ON GENETIC RISKS SCORES

Although elevated Lp(a) confers similar CVD risk across ancestries [58], median levels differ widely from ≈6 mg/dl in East Asians and Finns to ≈40–50 mg/dl in some African populations, reflecting substantial differences in trait distribution [59,60^▪^,61^▪^]. South Asians exhibit the second-highest median Lp(a) levels after Africans and about one in three individuals worldwide with Lp(a) more than 50 mg/dl is of South Asian descent [61^▪^]. The reason for elevated Lp(a) in South Asians remains unclear, as no higher frequency of LMW isoforms has been consistently observed, suggesting population-specific regulatory effects [61^▪^], similar to the higher frequency of the regulatory variant rs1800769 in Africans [25,62]. In contrast, a higher frequency of large isoforms explains the lower levels in Chinese [63^▪▪^,^64^].

SNP patterns and SNP-isoform associations differ markedly between ancestries [19]. For instance, rs3798220 was found to not tag short apo(a) isoforms or high Lp(a) in South Asians [65] or Chinese [53^▪▪^,^63^^▪▪^,^66^], and two small studies found also no association of rs3798220 with coronary artery disease in Iraqis and Iranians (recently reviewed in [60^▪^]). Conversely, rs10455872 is associated with CVD also in Middle East [60^▪^]. However, it is absent in Chinese [63^▪▪^,^66^] and very rare in South Asians [55].

A recent GWAS in more than 18 000 Chinese with follow-up in the UK Biobank provides very interesting insights into the genetics of Lp(a) in East Asia [63^▪▪^]. The two Chinese lead variants (rs192717255, rs73596816) explained 11.3 and 10.4% of Lp(a) variance [63^▪▪^], which is substantially less than the variance explained in Europeans by rs10455872 alone (24–29% [67,68]) or rs10455872 and rs3798220 together (36%[56]). In line with this, a 28-SNP score derived from Chinese explained only 10% variance in UK Biobank (would be ≈60% for a comparable European SNP score [69]) and adding Chinese SNPs to European SNPs scores, or vice versa, did not improve explained variance in the other ancestry. This highlights considerable differences in the genetic architecture of the Lp(a) trait between Chinese and Europeans, possibly also hinting towards a higher allelic heterogeneity in Chinese. However, the authors also replicated the association of *APOE* with Lp(a) observed previously in Europeans [67,68,70], confirming still shared biology despite divergent genetic patterns.

These strong ancestry-specific components limit the transferability of SNP-based genetic risk scores (GRS) for Lp(a) across populations [71] (in contrast to the KIV-2 copy number effect [51]), as noted by Privé *et al.* [72] who observed that the best Lp(a) GRS explains 66% in British, but only 40% in Iranians, 15% in Caribbeans, and 0% in Nigerians. As hardly any high-impact *LPA* variants for non-European groups are known, more diverse sequencing efforts are urgently required.

## PROGRESSES IN IDENTIFYING REGULATORS OUTSIDE OF THE *LPA* GENE

The role of regulators outside *LPA* and especially the identity of the Lp(a) receptor(s) remain largely unknown, with no single major receptor identified yet [17^▪▪^,^73^,^74^]. The LDL receptor (*LDLR*) is the leading candidate and has been shown to bind Lp(a) in vitro, but in-vivo evidence is inconsistent [17^▪▪^]. Some authors proposed that *LDLR* may contribute noticeably only under conditions of supraphysiological receptor expression and particularly low LDL-C, as achieved by statin therapy plus *PCSK9* inhibition [17^▪▪^,^75^].

Recently, Lp(a) internalization via macropinocytosis mediated by interaction of the apo(a) moiety with plasminogen receptors has been proposed [76], but in a recent follow-up work the macropinocytosis inhibitor imipramine did actually *stimulate* Lp(a) internalization, which was found to be due to upregulation of the plasminogen receptor PlgRKT and subsequent increased anchoring of Lp(a) to the cell membrane for S100A10- and Annexin A2-induced macropinocytosis [77^▪^]. Others noted, however, that hepatocytes are not known to rely on macropinocytosis and suggested that the true Lp(a) receptor remains unidentified [17^▪▪^,^78^^▪▪^]. In an effort to scale up the search for Lp(a) receptors in an agnostic way, Khan *et al.* [79^▪▪^] recently performed a genome-wide CRISPR knockout screening in HuH7 cells interrogating the effect of more than 19 000 genes on Lp(a) uptake. Although such an approach is inherently limited by the specific gene expression pattern of the used cell line, it is still noteworthy that it retrieved only *LDLR* and *MYLIP* (a negative regulator of LDLR) as significant positive, respectively negative determinants of Lp(a) uptake. Also relaxing the significance threshold brought up only further *LDLR* regulators, which is in line with a large GWAS that, among all proposed receptor candidates, found only an association signal in *LDLR -* but its effect on Lp(a) variance was only ≈1% that of rs14055872 [67]. Surely, the Lp(a) metabolism remains a conundrum.

Lambert and Boffa [78^▪▪^] recently noted that such endeavors assume that a discrete Lp(a) receptor analogous to the LDL–LDLR system exist. Given the recent evolutionary origin of Lp(a), no specific receptor may have co-evolved and Lp(a) uptake may involve multiple receptors with incidental affinity [78^▪▪^]. This would be consistent with the somewhat inconclusive biochemical data implicating many candidates and mechanisms to different extents. This would make identification of an “Lp(a) receptor” difficult and might even question its suitability as a drug target.

## CONCLUSION

The very reason for the existence of Lp(a) remains a mystery and the mutation patterns of the *LPA* gene have been even compared to those of a transcribed pseudogene [17^▪▪^,^80^], raising doubts about whether it really serves a physiological role. The forthcoming technological advances are poised to provide unprecedented insights into the variation patterns in *LPA* and the genetic architecture of Lp(a). Putting such complete genetic data into a formal evolutionary framework may finally help to answer whether Lp(a) is truly an adaptive trait or just an evolutionary byproduct, with important implications across the field. This may bring us one step closer to resolving one of the many riddles of that Lp(a) poses.

## Acknowledgements

*Given the focus on developments from the past 18 months and word count limitations, it was necessary to prioritize topics. The author regrets omitting numerous valuable contributions to the field and encourages readers to explore other excellent review articles offering complementary perspectives.*

*Figure 2 uses icons from Bioicons, NIAID NIH BIOART and svgrepo.com. Attributions Bioicons: Icon nanopore_sequencing icon by DBCLS (* *https://togotv.dbcls.jp/en/pics.html* *) is licensed under CC-BY 4.0 (* *http://creativecommons.org/licenses/by/4.0/* *). Icon img_sequencer_long_read05 by PacBio (* *www.pacb.com* *) is licensed under CC0 (* *https://creativecommons.org/publicdomain/zero/1.0/* *).*

*Attributions NIAID NIH BIOART: Icon Gene Mutation: NIAID Visual & Medical Arts. (10/7/2024), bioart.niaid.nih.gov/bioart/170. Icon Next Gen Sequencer: NIAID Visual & Medical Arts. (10/7/2024), bioart.niaid.nih.gov/bioart/386. Attribution Svgrepo.com: World map icon under MIT license,**https://www.svgrepo.com/svg/402581/world-map*.

### Financial support and sponsorship

*This research was funded in whole or in part by the Austrian Science Fund (FWF) 10.55776/PAT5152823 to S.C.*

### Conflicts of interest

*The author has received honoraria from Novartis AG (Basel) and Silence Therapeutics, PLC (London) for consulting activities related to Lp(a) genetics.*

## Supplementary Material

## REFERENCES AND RECOMMENDED READING

Papers of particular interest, published within the annual period of review, have been highlighted as:

▪ of special interest
▪▪ of outstanding interest

## References

[1] Kronenberg F Mora S Stroes ESG. Lipoprotein(a) in atherosclerotic cardiovascular disease and aortic stenosis: a European Atherosclerosis Society consensus statement. Eur Heart J 2022; 43:3925–3946. 36036785 10.1093/eurheartj/ehac361 PMC9639807

[2▪▪] Nordestgaard BG Langsted A. Lipoprotein(a) and cardiovascular disease. Lancet 2024; 404:1255–1264. 39278229 10.1016/S0140-6736(24)01308-4

[3▪] Anchouche K Baass A Thanassoulis G. Lp(a): a clinical review. Clin Biochem 2025; 137:110929. 40258460 10.1016/j.clinbiochem.2025.110929

[4] Patel AP Wang M Pirruccello JP. Lp(a) (Lipoprotein [a]) concentrations and incident atherosclerotic cardiovascular disease: new insights from a large National Biobank. Arterioscler Thromb Vasc Biol 2021; 41:465–474. 33115266 10.1161/ATVBAHA.120.315291 PMC7769893

[5▪▪] Kronenberg F Bedlington N Ademi Z. The Brussels International Declaration on Lipoprotein(a) Testing and Management. Atherosclerosis 2025; 406:119218. 40340180 10.1016/j.atherosclerosis.2025.119218

[6▪] Assini JM Boffa MB Koschinsky ML. The complex pro-atherosclerotic role of lipoprotein(a): a multiplicity of cellular targets. Curr Opin Lipidol 2025; 36:268–275. 40748007 10.1097/MOL.0000000000001000

[7] Björnson E Adiels M Taskinen MR. Lipoprotein(a) is markedly more atherogenic than LDL: an apolipoprotein B-based genetic analysis. J Am Coll Cardiol 2024; 83:385–395. 38233012 10.1016/j.jacc.2023.10.039 PMC7616706

[8▪] Mora S Kronenberg F. Lipoprotein(a). JAMA 2025; 333:1918–1919. 40272827 10.1001/jama.2025.2373

[9] Kamstrup PR Benn M Tybjaerg-Hansen A. Extreme lipoprotein(a) levels and risk of myocardial infarction in the general population: the Copenhagen City Heart Study. Circulation 2008; 117:176–184. 18086931 10.1161/CIRCULATIONAHA.107.715698

[10▪] Koschinsky ML Soffer DE Boffa MB. What's next for lipoprotein(a)? A national lipid association report from an expert panel discussion. J Clin Lipidol 2024; 18:e886–e892. 39299825 10.1016/j.jacl.2024.06.005

[11▪] Tsimikas S. Lipoprotein(a) in the year 2024: a look back and a look ahead. Arterioscler Thromb Vasc Biol 2024; 44:1485–1490. 38924439 10.1161/ATVBAHA.124.319483 PMC11210685

[12] Nestel P. Lipoprotein(a) removal still a mystery. J Am Heart Assoc 2019; 8:e011903. 30755058 10.1161/JAHA.118.011903 PMC6405664

[13] Erhart G Lamina C Lehtimäki T. Genetic factors explain a major fraction of the 50% lower lipoprotein(a) concentrations in Finns. Arterioscler Thromb Vasc Biol 2018; 38:1230–1241. 29567679 10.1161/ATVBAHA.118.310865 PMC5943067

[14] Waldeyer C Makarova N Zeller T. Lipoprotein(a) and the risk of cardiovascular disease in the European population: results from the BiomarCaRE consortium. Eur Heart J 2017; 38:2490–2498. 28449027 10.1093/eurheartj/ehx166 PMC5837491

[15] Kraft HG Köchl S Menzel HJ. The apolipoprotein (a) gene: a transcribed hypervariable locus controlling plasma lipoprotein (a) concentration. Hum Genet 1992; 90:220–230. 1336760 10.1007/BF00220066

[16] Boerwinkle E Leffert CC Lin J. Apolipoprotein(a) gene accounts for greater than 90% of the variation in plasma lipoprotein(a) concentrations. J Clin Invest 1992; 90:52–60. 1386087 10.1172/JCI115855 PMC443062

[17▪▪] Boffa MB Koschinsky ML. Lipoprotein(a) and cardiovascular disease. Biochem J 2024; 481:1277–1296. 39302109 10.1042/BCJ20240037 PMC11555715

[18] Coassin S Schönherr S Weissensteiner H. A comprehensive map of single-base polymorphisms in the hypervariable LPA kringle IV type 2 copy number variation region. J Lipid Res 2019; 60:186–199. 30413653 10.1194/jlr.M090381 PMC6314250

[19] Coassin S Kronenberg F. Lipoprotein(a) beyond the kringle IV repeat polymorphism: the complexity of genetic variation in the LPA gene. Atherosclerosis 2022; 349:17–35. 35606073 10.1016/j.atherosclerosis.2022.04.003 PMC7613587

[20] Kronenberg F Utermann G. Lipoprotein(a): resurrected by genetics. J Intern Med 2013; 273:6–30. 22998429 10.1111/j.1365-2796.2012.02592.x

[21] Perombelon YFN Soutar AK Knight BL. Variation in lipoprotein(a) concentration associated with different apolipoprotein(a) alleles. J Clin Invest 1994; 93:1481–1492. 8163653 10.1172/JCI117126 PMC294162

[22] Cohen JC Chiesa G Hobbs HH. Sequence polymorphisms in the apolipoprotein (a) gene. Evidence for dissociation between apolipoprotein(a) size and plasma lipoprotein(a) levels. J Clin Invest 1993; 91:1630–1636. 8473506 10.1172/JCI116370 PMC288140

[23] Coassin S Erhart G Weissensteiner H. A novel but frequent variant in LPA KIV-2 is associated with a pronounced Lp(a) and cardiovascular risk reduction. Eur Heart J 2017; 38:1823–1831. 28444229 10.1093/eurheartj/ehx174 PMC5837733

[24] Schachtl-Riess JF Kheirkhah A Grüneis R. Frequent LPA KIV-2 variants lower lipoprotein(a) concentrations and protect against coronary artery disease. J Am Coll Cardiol 2021; 78:437–449. 34325833 10.1016/j.jacc.2021.05.037 PMC7613585

[25] Mukamel RE Handsaker RE Sherman MA. Protein-coding repeat polymorphisms strongly shape diverse human phenotypes. Science 2021; 373:1499–1505. 34554798 10.1126/science.abg8289 PMC8549062

[26] Grüneis R Weissensteiner H Lamina C. The kringle IV type 2 domain variant 4925G>A causes the elusive association signal of the LPA pentanucleotide repeat. J Lipid Res 2022; 63:100306. 36309064 10.1016/j.jlr.2022.100306 PMC9700027

[27] Grüneis R Lamina C Di Maio S. The effect of LPA Thr3888Pro on lipoprotein(a) and coronary artery disease is modified by the LPA KIV-2 variant 4925G>A. Atherosclerosis 2022; 349:151–159. 35534298 10.1016/j.atherosclerosis.2022.04.023 PMC7613586

[28] Wagner J Olson ND Harris L. Curated variation benchmarks for challenging medically relevant autosomal genes. Nat Biotechnol 2022; 40:672–680. 35132260 10.1038/s41587-021-01158-1 PMC9117392

[29] Majidian S Agustinho DP Chin CS. Genomic variant benchmark: if you cannot measure it, you cannot improve it. Genome Biol 2023; 24:221. 37798733 10.1186/s13059-023-03061-1 PMC10552390

[30▪] Taylor DJ Eizenga JM Li Q. Beyond the Human Genome Project: the age of complete human genome sequences and pangenome references. Annu Rev Genomics Hum Genet 2024; 25:77–104. 38663087 10.1146/annurev-genom-021623-081639 PMC11451085

[31] Mahmoud M, Harting J, Corbitt H, *et al.* Closing the gap: solving complex medically relevant genes at scale. medRxiv 2024; doi: 10.1101/2024.03.14.24304179.

[32] Vollger MR Guitart X Dishuck PC. Segmental duplications and their variation in a complete human genome. Science 2022; 376:eabj6965. 35357917 10.1126/science.abj6965 PMC8979283

[33] Wagner J Olson ND Harris L. Benchmarking challenging small variants with linked and long reads. Cell Genom 2022; 2:100128. 36452119 10.1016/j.xgen.2022.100128 PMC9706577

[34] Chin C-S Behera S Khalak A. Multiscale analysis of pangenomes enables improved representation of genomic diversity for repetitive and clinically relevant genes. Nat Methods 2023; 20:1213–1221. 37365340 10.1038/s41592-023-01914-y PMC10406601

[35] Liao WW Asri M Ebler J. A draft human pangenome reference. Nature 2023; 617:312–324. 37165242 10.1038/s41586-023-05896-x PMC10172123

[36▪] Rausch T Marschall T Korbel JO. The impact of long-read sequencing on human population-scale genomics. Genome Res 2025; 35:593–598. 40228902 10.1101/gr.280120.124 PMC12047236

[37] Nurk S Koren S Rhie A. The complete sequence of a human genome. Science 2022; 376:44–53. 35357919 10.1126/science.abj6987 PMC9186530

[38▪] Di Maio S Zöscher P Weissensteiner H. Resolving intra-repeat variation in medically relevant VNTRs from short-read sequencing data using the cardiovascular risk gene LPA as a model. Genome Biol 2024; 25:167. 38926899 10.1186/s13059-024-03316-5 PMC11201333

[39] Chin C-S, Behera S, Metcalf GA, *et al.* A pan-genome approach to decipher variants in the highly complex tandem repeat of LPA. bioRxiv 2022:2022. 06.08.495395. DOI: 10.1101/2022.06.08.495395.

[40] Logsdon GA Ebert P Audano PA. Complex genetic variation in nearly complete human genomes. Nature 2025; 644:430–441. 40702183 10.1038/s41586-025-09140-6 PMC12350169

[41▪] Gustafson JA Gibson SB Damaraju N. High-coverage nanopore sequencing of samples from the 1000 Genomes Project to build a comprehensive catalog of human genetic variation. Genome Res 2024; 34:2061–2073. 39358015 10.1101/gr.279273.124 PMC11610458

[42] Gong J Sun H Wang K. Long-read sequencing of 945 Han individuals identifies structural variants associated with phenotypic diversity and disease susceptibility. Nat Commun 2025; 16:1494. 39929826 10.1038/s41467-025-56661-9 PMC11811171

[43▪] Schloissnig S Pani S Ebler J. Structural variation in 1,019 diverse humans based on long-read sequencing. Nature 2025; 644:442–452. 40702182 10.1038/s41586-025-09290-7 PMC12350158

[44▪] Hansen NF, Dwarshuis N, Ji HJ, *et al.* A complete diploid human genome benchmark for personalized genomics. bioRxiv 2025:2025.09.21.677443. DOI: 10.1101/2025.09.21.677443. 10.1016/j.cell.2026.06.016 PMC13456413 42561913

[45▪] Prodanov T, Plender EG, Seebohm G, *et al.* Locityper: targeted genotyping of complex polymorphic genes. bioRxiv 2025; 2024.05.03.592358. DOI: 10.1101/2024.05.03.592358. 10.1038/s41588-025-02362-4 PMC12597825 41107551

[46▪] Amstler S Streiter G Pfurtscheller C. Nanopore sequencing with unique molecular identifiers enables accurate mutation analysis and haplotyping in the complex lipoprotein(a) KIV-2 VNTR. Genome Med 2024; 16:117. 39380090 10.1186/s13073-024-01391-8 PMC11462820

[47] Barbieri G Cassioli G Kura A. Digital droplet PCR versus quantitative PCR for lipoprotein (a) kringle IV type 2 repeat polymorphism genetic characterization. J Clin Lab Anal 2024; 38:e24998. 38444303 10.1002/jcla.24998 PMC10959181

[48] Kronenberg F. Prediction of cardiovascular risk by Lp(a) concentrations or genetic variants within the LPA gene region. Clin Res Cardiol Suppl 2019; 14 (Suppl 1):5–12. 30859385 10.1007/s11789-019-00093-5

[49] Tsimikas S. In search of a physiological function of lipoprotein(a): causality of elevated Lp(a) levels and reduced incidence of type 2 diabetes. J Lipid Res 2018; 59:741–744. 29610122 10.1194/jlr.C085639 PMC5928428

[50] Gudbjartsson DF Thorgeirsson G Sulem P. Lipoprotein(a) concentration and risks of cardiovascular disease and diabetes. J Am Coll Cardiol 2019; 74:2982–2994. 31865966 10.1016/j.jacc.2019.10.019

[51] Telis N Dai H Waring A. Novel method for predicting Lp(a) from genomic testing identifies ASCVD risk across a diverse cohort. JACC Basic Transl Sci 2025; 10:101298. 40742365 10.1016/j.jacbts.2025.04.012 PMC12665494

[52▪] Betschart RO Koliopanos G Garg P. Comprehensive analysis of the genetic variation in the LPA gene from short-read sequencing. BioMed 2024; 4:156–170.

[53▪▪] Behera S Belyeu JR Chen X. Identification of allele-specific KIV-2 repeats and impact on Lp(a) measurements for cardiovascular disease risk. BMC Med Genomics 2024; 17:255. 39449055 10.1186/s12920-024-02024-0 PMC11515395

[54▪] Molitor C Labidi T Rimbert A. KILDA: identifying KIV-2 repeats from kmers. NAR Genom Bioinform 2025; 7:lqaf070. 40453649 10.1093/nargab/lqaf070 PMC12123407

[55] Lanktree MB Anand SS Yusuf S. Comprehensive analysis of genomic variation in the LPA locus and its relationship to plasma lipoprotein(a) in South Asians, Chinese, and European Caucasians. Circ Cardiovasc Genet 2010; 3:39–46. 20160194 10.1161/CIRCGENETICS.109.907642

[56] Clarke R Peden JF Hopewell JC. Genetic variants associated with Lp(a) lipoprotein level and coronary disease. N Engl J Med 2009; 361:2518–2528. 20032323 10.1056/NEJMoa0902604

[57] Eberle MA, Belyeu JR, Chen Xi. WIPO Patent application WO2023192942A1: copy number variant calling for lpa kiv-2 repeat. Illumina, Inc. World Intellectual Property Organization (WIPO), Geneva: Switzerland. 2023.

[58] Koschinsky ML Bajaj A Boffa MB. A focused update to the 2019 NLA scientific statement on use of lipoprotein(a) in clinical practice. J Clin Lipidol 2024; 18:e308–e319. 38565461 10.1016/j.jacl.2024.03.001

[59] Pare G Caku A McQueen M. Lipoprotein(a) levels and the risk of myocardial infarction among 7 ethnic groups. Circulation 2019; 139:1472–1482. 30667276 10.1161/CIRCULATIONAHA.118.034311

[60▪] El-Menyar A Khan NA Al Mahmeed W. Cardiovascular implications of lipoprotein(a) and its genetic variants: a critical review from the Middle East. JACC Asia 2025; 5:847–864. 40610121 10.1016/j.jacasi.2025.04.012 PMC12277198

[61▪] Patel D Koschinsky ML Agarwala A. Role of lipoprotein(a) in atherosclerotic cardiovascular disease in South Asian individuals. J Am Heart Assoc 2025; 14:eJAHA2024040361T. 10.1161/JAHA.124.040361 PMC13055799 40654252

[62] Chretien JP Coresh J Berthier-Schaad Y. Three single-nucleotide polymorphisms in LPA account for most of the increase in lipoprotein(a) level elevation in African Americans compared with European Americans. J Med Genet 2006; 43:917–923. 16840570 10.1136/jmg.2006.042119 PMC2563202

[63▪▪] Clarke R Wright N Lin K. Causal relevance of Lp(a) for coronary heart disease and stroke types in East Asian and European ancestry populations: a Mendelian Randomization study. Circulation 2025; 151:1699–1711. 40297899 10.1161/CIRCULATIONAHA.124.072086 PMC12165552

[64] Sandholzer C Hallman DM Saha N. Effects of the apolipoprotein(a) size polymorphism on the lipoprotein(a) concentration in 7 ethnic groups. Hum Genet 1991; 86:607–614. 2026424 10.1007/BF00201550

[65] Khalifa M Noureen A Ertelthalner K. Lack of association of rs3798220 with small apolipoprotein(a) isoforms and high lipoprotein(a) levels in East and Southeast Asians. Atherosclerosis 2015; 242:521–528. 26302166 10.1016/j.atherosclerosis.2015.07.015

[66] Li J Ma B Fang Q. Lipoprotein(a) molar concentrations rather than genetic variants better predict coronary artery disease risk and severity in Han Chinese population. Lipids Health Dis 2025; 24:49. 39953584 10.1186/s12944-025-02467-z PMC11827131

[67] Said MA Yeung MW van de Vegte YJ. Genome-wide association study and identification of a protective missense variant on lipoprotein(a) concentration. Arterioscler Thromb Vasc Biol 2021; 41:1792–1800. 33730874 10.1161/ATVBAHA.120.315300

[68] Hoekstra M Chen HY Rong J. Genome-wide association study highlights APOH as a novel locus for lipoprotein(a) levels. Arterioscler Thromb Vasc Biol 2021; 41:458–464. 33115273 10.1161/ATVBAHA.120.314965 PMC7769958

[69] Trinder M Uddin MM Finneran P. Clinical utility of lipoprotein(a) and LPA genetic risk score in risk prediction of incident atherosclerotic cardiovascular disease. JAMA Cardiol 2020; 6:287–295. 10.1001/jamacardio.2020.5398 PMC7539232 33021622

[70] Mack S Coassin S Rueedi R. A genome-wide association meta-analysis on lipoprotein (a) concentrations adjusted for apolipoprotein (a) isoforms. J Lipid Res 2017; 58:1834–1844. 28512139 10.1194/jlr.M076232 PMC5580897

[71] Lee MP Dimos SF Raffield LM. Ancestral diversity in lipoprotein(a) studies helps address evidence gaps. Open Heart 2023; 10:e002382. 37648373 10.1136/openhrt-2023-002382 PMC10471864

[72] Privé F Aschard H Carmi S. Portability of 245 polygenic scores when derived from the UK Biobank and applied to 9 ancestry groups from the same cohort. Am J Hum Genet 2022; 109:12–23. 34995502 10.1016/j.ajhg.2021.11.008 PMC8764121

[73] Chemello K Chan DC Lambert G. Recent advances in demystifying the metabolism of lipoprotein(a). Atherosclerosis 2022; 349:82–91. 35606080 10.1016/j.atherosclerosis.2022.04.002

[74] McCormick SPA Schneider WJ. Lipoprotein(a) catabolism: a case of multiple receptors. Pathology 2019; 51:155–164. 30595508 10.1016/j.pathol.2018.11.003

[75] Lambert G Thedrez A Croyal M. The complexity of lipoprotein (a) lowering by PCSK9 monoclonal antibodies. Clin Sci (Lond) 2017; 131:261–268. 28108631 10.1042/CS20160403

[76] Siddiqui H Deo N Rutledge MT. Plasminogen receptors promote lipoprotein(a) uptake by enhancing surface binding and facilitating macropinocytosis. Arterioscler Thromb Vasc Biol 2023; 43:1851–1866. 37589135 10.1161/ATVBAHA.123.319344 PMC10521804

[77▪] Deo N Siddiqui H Peppercorn K. Antidepressants stimulate lipoprotein(a) macropinocytosis via serotonin-enhanced cell surface binding. J Lipid Res 2025; 66:100889. 40865611 10.1016/j.jlr.2025.100889 PMC12482634

[78▪▪] Lambert GC Boffa MB. Why has the “Lp(a) receptor” proven so elusive? Atherosclerosis 2025; 404:119189. 40215895 10.1016/j.atherosclerosis.2025.119189

[79▪▪] Khan TG Bragazzi Cunha J Raut C. Functional interrogation of cellular Lp(a) uptake by genome-scale CRISPR screening. Atherosclerosis 2025; 403:119174. 40174266 10.1016/j.atherosclerosis.2025.119174 PMC12011201

[80] Koschinsky ML Boffa MB. Genetics to the rescue: sophisticated approaches provide critical insights into the determination of Lp(a) levels. J Am Coll Cardiol 2021; 78:450–452. 34325834 10.1016/j.jacc.2021.06.004

