# Supplementary material for: Recent advances in understanding the spectrum of genetic determinants of lipoprotein(a) levels
Source: Curr Opin Lipidol. 2026 Feb 6;37(2):65–72. doi: 10.1097/MOL.0000000000001030 (PMC12978723; doi:10.1097/MOL.0000000000001030)
Supplement: Supplementary file 1 [file colip-37-65-s001.docx]

Supplementary materials to

**Recent advances in understanding**

**the spectrum of genetic determinants of Lipoprotein(a) levels**

Stefan Coassin

**Supplementary Table 1. Features of the *LPA* gene that complicate its analysis by conventional short-read sequencing and genotyping.**

| **Feature** | **Description** | **Issue(s) caused** |
| --- | --- | --- |
| KIV-2 repeat | Up to ≈40 repeats of a 5.6 kb large region with >98% homology to each other. | Sequencing reads cannot be mapped uniquely to the reference genome and are therefore discarded by analysis algorithms. The signal of variants present only in one or some repeats is diluted. |
| KIV-2 subtypes  [1,2,3] | At least three subtypes of KIV-2 (-A, -B, -C) defined by specific SNPs in the introns and in KIV-2 exon 1 exist, but not every individual carries them and both their frequency and their abundance within the KIV-2 units varies between ancestries. Moreover, KIV-2B exon 1 is identical to KIV-3 exon 1. The latter is, however, present in every individual. | As KIV-3 units is present in every individual, but not KIV-2B units, KIV-3 reads are partially mistaken as KIV-2B reads and depending on whether the individual carries bona-fide KIV-2B units or not, different kind of sequencing artifacts can arise (see references [3] and [4]. |
| High homology across all KIV domains | All KIV domains are highly homologous to each other with >80% per‑base identify between exon 1 of the KIV domains and >70% between exon 2 of the KIV domains. Especially KIV-1 to KIV-4 are highly homologous to each other, as well as KIV-6 to KIV-8[3]. | When sequencing data contains reads from all KIV units (like whole-genome or whole-exome data), these reads can map to the wrong kringle and natural differences between the kringle sequences create spurious variant calls. This is exacerbated if such data is aligned only to one kringle as done in the “*LPA* batch sequencing” strategy that is commonly used to call variants within the KIV-2 region.[3,4]. We have recently described a variant calling tool that alleviates this issue and doubles KIV-2 variant calling accuracy[4]. |
| Intronic long interspersed nuclear elements (LINEs) | Sequences derived from LINE-1 retrotransposons are found in every KIV intron[5] | Short-read mapping to the introns can be ambiguous if LINE sequences are contained. The same applies to genotyping using short amplicons and probes like in TaqMan assays. For example, the strongly Lp(a)‑increasing SNP rs140570886[6,7,8] is located in such a LINE-1 element and is, therefore, difficult to genotype using TaqMan assays. |
| *LPAL2* pseudogene | An expressed *LPA* pseudogene named *LPAL2* of is located upstream of *LPA. LPAL2* contains sequences homologous to KIV-2, KIV-9 and KIV-10, as well as segments of KIV-3 and KV. | Reads from *LPAL2* may map to *LPA* and vice versa, which induces spurious variant calls. The pseudogene expresses two transcripts differing in the last exon. Importantly, *LPAL2* shows similar liver expression levels as *LPA (*according to GTEx data[9]*)*, but, in contrast to *LPA*, it is expressed to some degree also in many other tissue. This could confound mRNA-based Lp(a) studies*.* |
| *PLG* and *PLG* pseudogenes | *LPA* originates from plasminogen and retains some homology to parts of this gene, especially in the promoter region. This applies also to the plasminogen pseudogenes *PLGLA*, *PLGLB1*, and *PLGLB2*. | While the coding sequences of *LPA* and *PLG*, respectively its pseudogenes, are sufficiently different to allow unique read mapping, some non-coding sequences and the promoter regions share various short segments of high similarity that can complicate PCR assay design. |

**References**

1. McLean JW, Tomlinson JE, Kuang WJ, et al. **cDNA sequence of human apolipoprotein(a) is homologous to plasminogen.** Nature 1987; 330:132-137

2. Parson W, Kraft HG, Niederstätter H, et al. **A common nonsense mutation in the repetitive Kringle IV-2 domain of human apolipoprotein(a) results in a truncated protein and low plasma Lp(a).** Hum Mutat 2004; 24:474-80

3. Coassin S, Schönherr S, Weissensteiner H, et al. **A comprehensive map of single-base polymorphisms in the hypervariable LPA kringle IV type 2 copy number variation region.** J Lipid Res 2019; 60:186-199

4. Di Maio S, Zöscher P, Weissensteiner H, et al. **Resolving intra-repeat variation in medically relevant VNTRs from short-read sequencing data using the cardiovascular risk gene LPA as a model.** Genome Biol 2024; 25:167

5. Li X, Liu N. **Advances in understanding LINE-1 regulation and function in the human genome.** Trends Genet 2025; 41:577-589

6. Zeng L, Moser S, Mirza-Schreiber N, et al. **Cis-epistasis at the LPA locus and risk of cardiovascular diseases.** Cardiovasc Res 2022; 118:1088-1102

7. Mack S, Coassin S, Rueedi R, et al. **A genome-wide association meta-analysis on lipoprotein (a) concentrations adjusted for apolipoprotein (a) isoforms.** J Lipid Res 2017; 58:1834-1844

8. Trinder M, Uddin MM, Finneran P, et al. **Clinical Utility of Lipoprotein(a) and LPA Genetic Risk Score in Risk Prediction of Incident Atherosclerotic Cardiovascular Disease.** JAMA Cardiol 2020; 6:1-9

9. Consortium G. **The GTEx Consortium atlas of genetic regulatory effects across human tissues.** Science 2020; 369:1318-1330
